# Education in consultation: A comparative survey-based assessment of perceived educational effectiveness within a university and community-based internal medicine residency

**DOI:** 10.15694/mep.2017.000153

**Published:** 2017-09-04

**Authors:** Robert Rope, Sylvia Bereknyei Merrell, Margaret Stedman, Brian Young

**Affiliations:** 1 Oregon Health and Science University Division of Nephrology and Hypertension; 2 Stanford University School of Medicine Division of General Medical Disciplines; 3 Stanford University School of Medicine Division of Nephrology; 4 University of California at Davis School of Medicine Division of Nephrology

**Keywords:** Medical education-graduate, consultation, survey research

## Abstract

This article was migrated. The article was marked as recommended.

**Background:** Education in consultation is a potentially valuable, but understudied, element of medical education. Inpatient consultation is an opportunity for significant subspecialist contact for resident trainees and an avenue for improving their knowledge and patient care across content areas. We evaluated the perceived educational effectiveness of education in consultation among internal medicine residents, within a university and a community-based program, as well as the role of barriers in medical training that may limit education.

**Methods:** We used a web-based survey expanded from a previously published survey consisting of 12 questions, including one free-response, on education in consultation. Data were analyzed descriptively and qualitatively. We surveyed residents from two internal medicine programs in 2016. One within a large university-based hospital and the second within a smaller community-based safety-net hospital.

**Results:** 91/198 (46%) of residents responded. Overall results from both programs were similar despite their structural differences. Residents viewed education in consultation as a priority and the majority felt it was at least moderately effective but underutilized. Importantly, educational interactions are largely dependent on outreach from residents. While in-person teaching interactions were the most effective, key barriers to these interactions include a lack of time, difficulty locating residents, and the perception of residents as being too busy.

**Conclusions:** Inpatient consultation offers a unique opportunity for specialist-led education for internal medicine residents. It is potentially effective but constrained extensively in modern training environments. Interventions aimed at emphasizing education in consultation within fellowships and residencies, increasing in-person resident-specialist interactions, and addressing structural barriers, may improve resident knowledge across specialties and strengthen patient care.

## Introduction

Inpatient consultation is a critical but understudied educational resource in medical training. While education is heralded as a key element in consultation, guidance for its implementation is lacking ( Goldman, Lee, & Rudd, 1983; E. M. Miloslavsky, Boyer, Winn, Stafford, & McSparron, 2016). Nonetheless, consultations are an opportunity for substantive contact between resident trainees and subspecialists, often fellows in training themselves. Subspecialist fellows are often valuable teachers for trainees at all levels, with a keen understanding of the educational needs and challenges of residency, as well as recent experience teaching residents. Furthermore **,** fellows often find enjoyment in teaching and often wish to improve their teaching skills ( E. Miloslavsky & Puig, 2014; Richards, Kelly, Fessler, & Roberts, 2013).

Maximizing the educational effectiveness of consultation is a worthy goal, and improvements may not only affect consultation quality and clinical care but may also influence future specialty career choices. However, there are significant challenges to consultative education, including shortened in-person contact, which limits learner assessment and the ability of a consultant to connect with learners; the lack of confidence that accompanies early subspecialty training; and the increasing pressures of documentation, patient experience, and safety ( E. M. Miloslavsky, Boyer, et al., 2016). Shorter hospital stays and duty-hour restrictions heighten these challenges. In addition, while training in teaching has expanded within residency, similar training in fellowship lags behind ( E. M. Miloslavsky, Boyer, et al., 2016).

One prior survey evaluated the educational effectiveness of inpatient consultation within a university-based internal medicine residency (E. Miloslavsky & Puig, 2014). While perceived as educational, consultations were an underutilized educational opportunity limited by physical and time constraints. One issue not addressed by this study was the potential effect of different training environments on this education. In order to increase diversity in trainee experience, many subspecialty programs send fellows to rotate at clinical sites outside of the sponsoring institution, such as community-based academic hospitals. This current study further characterizes the educational nature of inpatient consultation and includes assessments within residency programs at a community-based as well as a university hospital.

## Methods

We surveyed internal medicine (IM) residents, as well as transitional residents completing an IM preliminary year. We developed questions from two sources: 1) adapting questions from a published survey with permission, and 2) the de-novo creation of questions based on our local training environments (E. Miloslavsky & Puig, 2014). The complete survey is the appendix. We distributed the survey to residents from two IM residency programs in the fourth quarter of the 2015-2016 academic year. The programs were a university hospital, Stanford University Hospital (SUH), and a community-based, safety-net hospital, Santa Clara Valley Medical Center (SCMVC). SUH fellows serve on consultation services at both SUH and SCVMC. Residents were instructed to consider teaching interactions in the process of consultation while on the requesting service and not when rotating on a consulting team. Participants completed the survey online through Qualtrics ( Qualtrics, 2015). We recruited participants at both programs via flyers (containing a survey hyperlink and quick response code) and word-of-mouth. We also distributed the survey via email at SCVMC. Participating residents received a five-dollar coffee gift card.

Results were re-weighted based on gender and class year to adjust for differences between the sample and source population. We compared results between groups using the Rao-Scott Chi-Square test which adjusts for survey weights. In cases of sparse results, we collapsed categories. We estimated weighted Pearson correlation coefficients to measure correlations between questions. Data were analyzed using Stata ( StataCorp, 2015). We collected free-text responses from participants to better understand the educational factors associated with consultation. Qualitative data was analyzed via an inductive, thematic approach, adjudicated amongst team members, with a final generation of representative common themes based on the content of the responses ( Miles, Huberman, & Saldana, 2014). This study was approved by the Institutional Review Boards of both SUH (IRB Study 36873) and SCVMC (IRB Study 16-002). Participants received an information sheet prior to taking the survey and completion of the survey was viewed as acknowledgment of informed consent.

## Results

The overall response rate was 91/198 (46%) with response rates of 42/119 (35%) for SUH and 49/79 (62%) for SCVMC. Demographics of the participants and their respective programs are shown in Table 1. Interns (PGY-1) responded more frequently at both sites, while respondents’ gender aligned with their programs.

**Figure:**
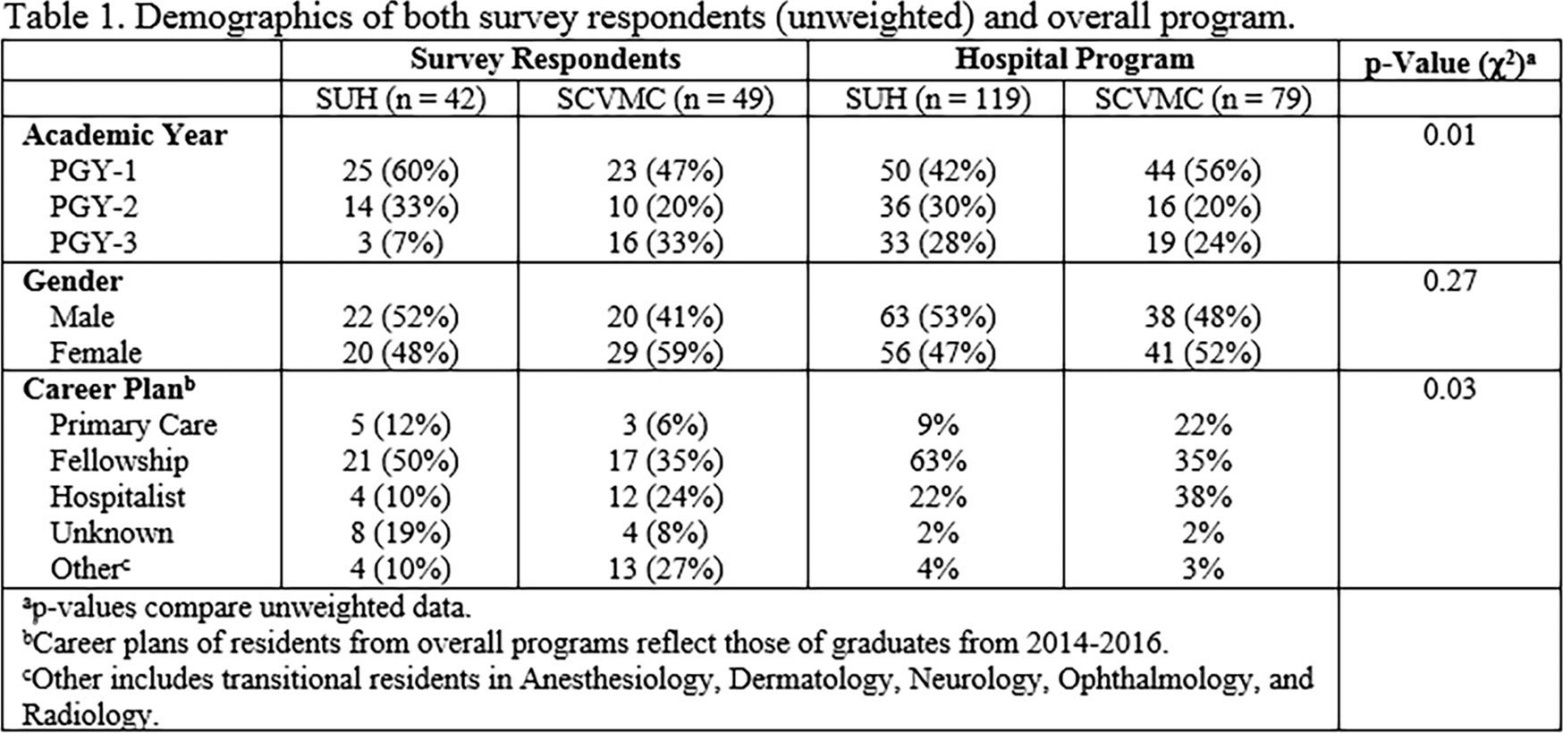


### Educational Effectiveness of Consultation

**Figure 1.**
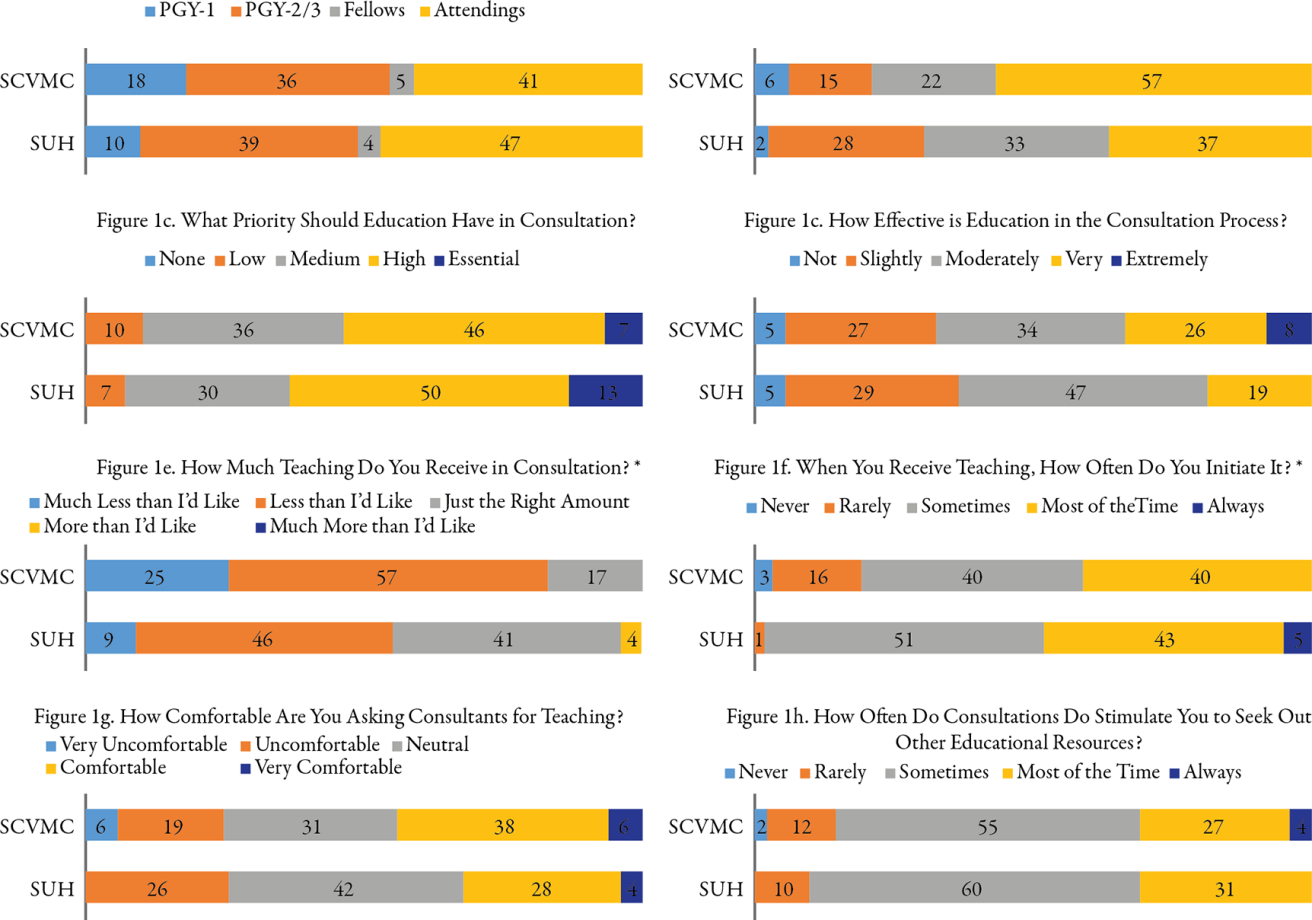
Responses to questions about education in consultation. Numbers represent percentage of respondents. * indicates p < 0.05 for comparison between programs.

Figures 1a-h show responses to questions regarding education in consultation. Residents obtain the most teaching from upper-level residents and attendings, though 22-33% receive the most effective teaching from fellows ( Figures 1a-1b). Over 90% of residents felt that education should be at least a medium, if not a high or essential, priority in consultation ( Figure 1c, SCVMC 90%, SUH 93%). However, while consultation is at least moderately effective for most residents, over half receive less teaching than desired ( Figures 1d-1e). In addition, residents, and not fellows, initiate most educational interactions, with the majority of residents less than comfortable initiating contact ( Figures 1f-g). Resident comfort in asking for teaching was positively correlated with the quantity of teaching received in consultation (p = 0.01) and the perceived effectiveness of that education (p < 0.01). Residents from SUH were more likely to initiate teaching interactions within consultation ( Figure 1f, p = 0.02) while those from SCVMC were more likely to receive less teaching than desired ( Figure 1e, p = 0.02). The remaining survey questions did not show differences between programs (p > 0.05). Lastly, educational interactions in consultations stimulate residents to seek additional educational resources, at least some of the time, if not most ( Figure 1h).

### Teaching Tools and Barriers to Education

**Figure 2.**
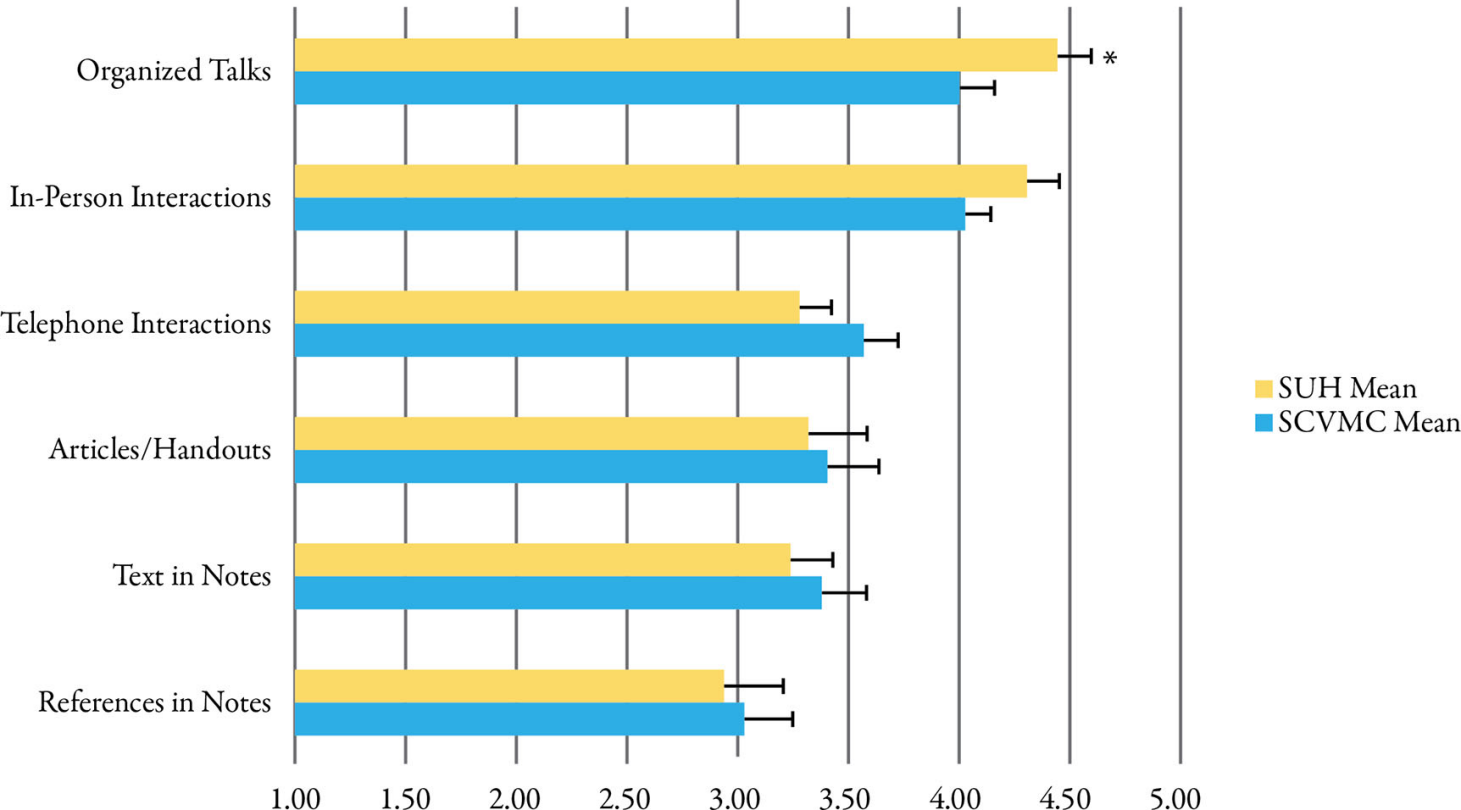
Perceived effectiveness of teaching tools in consultation from not effective (1) to extremely effective (5). Data represent means (SE). * indicates p < 0.05 for comparison between programs.

Respondents reported that in-person interactions and organized talks were the most effective teaching tools while consult notes were considerably less effective ( Figure 2). Organized talks were perceived as more effective at SUH (p < 0.05) however are infrequently used. Residents prefer to learn recommendations via telephone or in-person (73% SCVMC, 89% SUH, NS).

**Figure 3.**
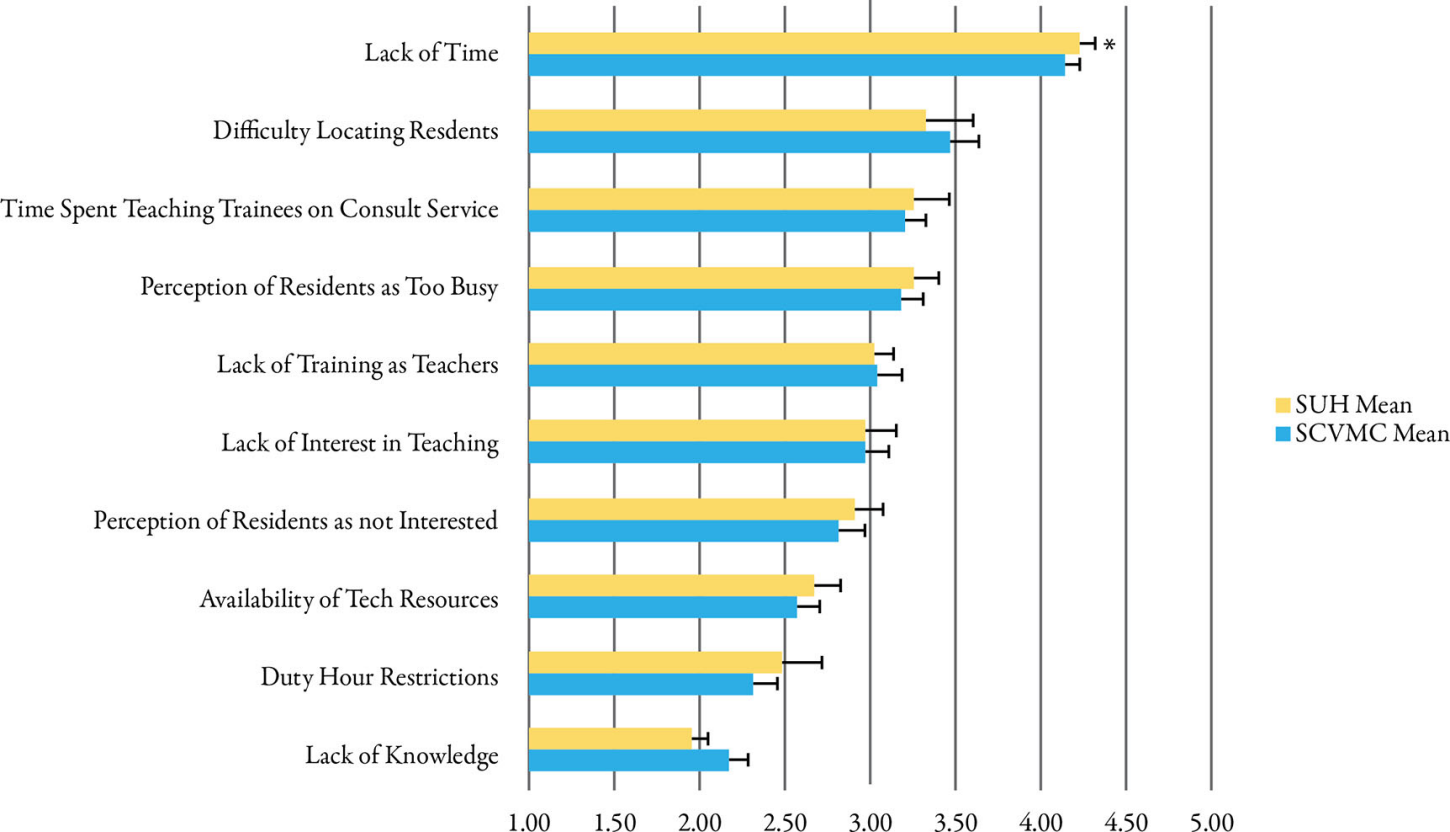
Resident agreement with potential barriers to teaching in consultation from strongly disagree (1), neither agree nor disagree (3), to strongly agree (5). Data represent means (SE). * indicates p < 0.05 for comparison between programs.

Resident assessments of potential barriers to teaching in consultation are shown in Figure 3. Key barriers included a lack of time, difficulty in physically locating residents, competing demands with learners on the consult service, and the perception of residents as being too busy. Resident interest, competing online resources, duty-hour restrictions, and lack of knowledge were not considered serious obstacles. Lack of time was perceived as a slightly more significant barrier at SUH (p < 0.05), despite the fact that education in consultation may be more predicated on resident outreach to fellows as noted above.

Overall, 26/91 respondents (29%) answered the free-response question: “Do you have any thoughts you would like us to know about the education process within inpatient consultation?” Qualitative themes were similar between programs and are listed in Table 2 with representative quotes. These themes included: 1) desire for education from consultants and the potential for consultations to increase resident self-sufficiency; 2) preference for cordial one-on-one communication; and 3) challenges to education, including limitations to fellows’ time and a lack of physical spaces for interactions. Residents highlighted the importance of verbal communication which is crucial for clarifying recommendations and generating educational interactions. Time and an inability to physically locate residents limit communication and education. Several residents remarked they did not learn effectively from notes which are often untimely, limited in explanations, and designed for documentation requirements rather than effective communication.

**Table 2.** Themes and representative quotes from the open-response question regarding education in consultation.

Theme	Representative Quote
Desire for education from Fellows and Potential to Increase ResidentSelf-Sufficiency	• “I love when I get teaching in phone or in person because if I learn it well then maybe I don’t have to consult you in the future.” (SCVMC, PGY-2) • “The more education the better! Teach more and we may not have to ask for help as often.” (SUH, PGY-2) • “I feel fellows have the best chance of teaching me well because they are close enough to remember what it was like to not quite understand the details, but more advanced than senior residents so they have an adequate understanding.” (SUH, PGY-1)
Preference for Cordial One-on-One Verbal Communication	• “I like when consultants come by and tell us their thoughts and I end up being able to really appreciate and absorb their recs [sic]. Sometimes fellows never actually get back to me with their thoughts, they just see the patient, drop a note, and I never get to talk to them. It is in these instances when I do not seek out their teaching because they have projected to me that they do not have time to talk to me directly.” (SUH, PGY-1) • “The best experiences I’ve had (and the most I’ve learned) during my interactions with fellows on consultation are the times I ask specific questions when discussing the patient either in-person or on the phone. I usually find that fellows are more than happy to elaborate and teach.” (SCVMC, PGY-2) • “In person teaching or telephone calls are most useful so that I can ask questions when they give me their recommendations.” (SCVMC, PGY-3) • “Most interactions with fellows are short or unpleasant because they spend significant time trying to block the pt [sic].” (SCVMC, PGY-1) • “Part of it is the relationship that is established with the consultant. Those that are approachable I’m much more comfortable asking probing questions and learning from.... Those that set up a hostile ‘defend your consult...attitude,’ I’m less likely to ask questions of.” (SUH, PGY-1)
Lack of Fellow Time and Educational Spaces are Challenges to Education	*Limited Fellow Time (Fellows Too Busy)* • “Fellows seem way too overworked. They’re all bright and nice people, but I can tell on the phone that they are overworked as most are abrupt on the phone or write very short notes that don’t explain their thought process.” (SCVMC, PGY-1) • “Usually [there is] not enough time to educate; fellows are pressed for consults and notes while on the other hand, it is difficult to get a hold of the teams in person or even on the phone to have adequate conversations.” (SCMVC, PGY-1) *Lack of Interaction and Private Spaces for Education (Such as team rooms or finding fellows)* • “We do not have team rooms at [SCVMC] which makes in-person teaching challenging, and it is difficult to find teams when they are rounding which would be a great time for quick teaching.” (SCVMC, PGY-2) • “One of the things I experienced in [SUH] was having team rooms. With team rooms, some consultants would just drop by and verbally give recs [sic]. It gave us an opportunity to ask questions directly and for them to give us their reasoning. I haven’t called a consultant after reading their note to ask any questions.” (SCVMC, PGY-1)

## Discussion

Education is considered a key element of consultation though the literature has limited direction for interested clinicians ( Goldman et al., 1983). This survey demonstrates that education by consultants in the residency setting is valuable but there is ample room for improvement.

Overall, residents perceive consultations to be an effective means of education. Though they perform less than 5% of primary teaching, fellows were identified by over 25% of residents as the most effective teachers, implying that the educational value of consultation may be out of proportion to the time spent. Consultation also stimulates self-directed learning by residents, which can expand the educational impact of an individual consultation.

However, effective education is less frequent than what residents would like and often depends on residents initiating the interaction. While nearly half of residents initiate teaching interactions “most” or “all of the time,” less than half felt comfortable in this role. Residents less comfortable with reaching out were less likely to receive effective education. Overall, SVCMC residents were less likely to initiate interactions and more likely to feel undereducated compared to SUH residents. While the reasons for this discrepancy are unclear, fellows working at both hospitals may prioritize education more during rotations within their main sponsoring SUH institution versus the affiliated SCVMC. Alternatively, interactions between residents and fellows may be more limited at SCVMC due to institutional or resident characteristics, which could negatively affect trainee comfort in communication. For instance, although most residents prefer learning consultation recommendations through telephone or in-person contact, residents at SCVMC may be less likely to prefer this type of communication. This is despite a lack of time being felt to be a more significant barrier at SUH than SCVMC. Given these concerns, further comparison of fellow teaching priorities between sites is required. If the individual clinical site does effect consultant teaching, this could be addressed by fellowship programs evaluating individual rotation characteristics and emphasizing the importance of education across all programs involved in their clinical training.

In consultation, verbal interaction is highly important, as reading a note does not allow for interaction and questioning of the consultant for deeper understanding. These results align with previously published data that physicians prefer direct verbal communication, rather than notes or text pages, as the primary communication tool ( E. Miloslavsky & Puig, 2014; Salerno, Hurst, Halvorson, & Mercado, 2007). Given these concerns, exploring ways to improve the consultant-resident relationship, particularly at clinical sites with graduate medical programs that are not from the main sponsoring institution, is paramount to improving resident education.

Overall, perceived barriers to effective education centered on systemic challenges (a lack of time to teach and inability to physically locate residents) and perception challenges (residents perceive that consultants view them as too busy). Time constraints were a recurrent theme, though residents did not specifically emphasize duty-hour restrictions as a barrier. It is notable that there were no substantial differences in these barriers between a large university program and a smaller community program, implying that these challenges are pervasive in training programs regardless of hospital setting.

Consult notes, in general, were not viewed as an effective educational tool. Thus, time spent formulating notes with textbook style commentaries and references may not be efficient in delivering educational content. Similarly, prior surveys have shown that only a minority of physicians view literature references as useful educational tools ( Salerno et al., 2007). Given these concerns, a laudable goal for consultants should be increasing time for direct communication and reducing time spent at a computer. Addressing the inherent inefficiencies in consult note-writing (e.g. repeating the work of others) as well as disconnecting notes from billing would go a long way towards achieving this goal.

Beyond changing our approach to note-writing, improving the educational effectiveness of consultations will require institutional emphasis within the training process ( Dotters-Katz, Hargett, Zaas, & Criscione-Schreiber, 2016). Divisions that emphasize education through their philosophies, expectations, and curricula motivate their fellows to teach ( E. M. Miloslavsky, McSparron, Richards, Puig, & Sullivan, 2015). Though curricula for faculty and resident clinician educators have been successfully developed, programs for fellows are limited ( Heflin, Pinheiro, Kaminetzky, & McNeill, 2009; Hill, Yu, Barrow, & Hattie, 2009; E. M. Miloslavsky, Boyer, et al., 2016; Richards, McCallister, & Lenz, 2016; Rosenbaum et al., 2011; Smith, McCormick, & Huang, 2014; Tofil et al., 2014). Curricula specific to teaching in consultation are necessary for fellowships given their unique challenges. These challenges include time constraints in modern medicine, shortened contact time between primary and consulting teams, and changing educational priorities when moving from generalist to sub-specialist training ( E. M. Miloslavsky, Boyer, et al., 2016; E. M. Miloslavsky et al., 2015). One initial study demonstrated the effectiveness of a “Fellow As Clinical Teacher” curriculum in improving participant skills in structured teaching exercises as well as fellow interest in teaching ( E. M. Miloslavsky, Criscione-Schreiber, et al., 2016). This group also recently showed that outreach to residents on primary teams, aimed at improving the quality of consult questions and encouraging residents to ask fellows for in-person teaching, successfully increased in-person teaching interactions during consultation (Gupta, Alladina, Heaton, & Miloslavsky, 2016). These educational innovations and emphasis may help address our study’s findings that one-on-one interactive education is desired but underutilized.

This study has several limitations, most notably that only two programs were surveyed. However, the consistency in results between the large university-based and smaller community-based programs, as well as with previously published results, speak to the generalizability of the results ( E. Miloslavsky & Puig, 2014). Despite this, the size of the study may limit appreciation of finer differences between the programs. Importantly, correlations between answers did not significantly change after re-weighting the data to adjust for differences in gender and academic year between the respondents and their overall residency populations. Although multiple recruitment strategies were used to mitigate the notoriously low response rates in studies of healthcare professionals, there was a lower response rate at SUH likely due to the lack of an e-mail distribution platform for the survey. However, the overall response rate is largely consistent with average provider response rates in the modern medical literature ( Cho, Johnson, & Vangeest, 2013). Lastly, this study focuses on the experiences of residents and not fellows, leaving part of the teaching dyad unexplored.

CONCLUSIONS

Education in the consultation process holds promise but is underutilized and has room for improvement. Aside from tackling larger structural issues, such as provider workloads and documentation requirements, consultation services could improve their educational impact through prioritization of in-person education during consultations or providing fellow training in teaching. In addition, increasing our understanding of the impact of resident-fellow personal interactions, and the effect of systemic influences across different training environments, will likely enable further improvements in consultation education. As such, further studies are needed to characterize the resident-fellow educational dyad, especially from the fellow perspective, and to evaluate additional solutions to these barriers.

## Take Home Messages

• Consultation is a valued but underutilized source of education for trainees.
• Educational interactions largely depend on outreach from trainees to consultants.
• In-person interactions are most effective; however, time and physical constraints limit them.
• Interventions to emphasize education in consultation within organizations and increase in-person interactions are needed.
• Curricula covering challenges in consultation may improve the educational effectiveness of consultant trainees.

## Notes On Contributors

Robert Rope designed and implemented the study and served as the primary author. Dr. Rope was a nephrology fellow at Stanford University during the study. He is now a practicing nephrologist at Oregon Health and Science University as well as Associate Program Director for the nephrology fellowship.

Sylvia Bereknyei Merrellcontributed to the development of the study and manuscript. Dr. Bereknyei Merrell is an education scholar and researcher in the Stanford University Division of General Medical Disciplines as well as a faculty leader of the Rathmann Medical Education Fellowship.

Margaret R. Stedman performed statistical analyses and contributed to the manuscript. Dr. Stedman is an ongoing biostatistician within the Stanford University Division of Nephrology.

Brian Young contributed to the development of the study and manuscript. Dr. Young was a practicing nephrologist within the Santa Clara Valley Medical Center Division of Nephrology as well as Associate Program Director for the internal medicine residency during the study. Currently, he is a practicing nephrologist at the University of California at Davis.

## Appendices

Education in Consultation Survey

From which of the following people do you receive the most teaching in terms of time?

a. PGY-1 (Intern)
b. PGY-2/3 (Residents)
c. Fellows
d. Attendings

From which of the following do you receive the most effective teaching?

a. PGY-1 (Intern)
b. PGY-2/3 (Residents)
c. Fellows
d. Attendings

How much teaching do you receive from clinical fellows when they consult on your patients?

a. Much less than I’d like
b. Less than I’d like
c. Just the right amount
d. More than I’d like
e. Much more than I’d like

When you do receive teaching from fellows, what percentage of the time do you feel you initiated the interaction?

a. Never
b. Rarely
c. Some of the time
d. Most of the time
e. Always

How comfortable do you feel asking consultant fellows for teaching?

a. Very Uncomfortable
b. Uncomfortable
c. Neutral
d. Comfortable
e. Very Comfortable

Based on your overall experience in residency, how effective is the consultation process in educating you?

a. Not Effective
b. Slightly Effective
c. Moderately Effective
d. Very Effective
e. Extremely Effective

What priority level do you feel consultants should place on education of the team requesting the consult?

a. Not a Priority
b. Low Priority
c. Medium Priority
d. High Priority
e. Essential Priority

What is your preferred method of learning from consultant fellows about their rationale and recommendations for the case?

a. Text Page
b. Telephone
c. In-Person
d. Consult Notes

How often do you feel a consultant’s discussion or recommendations stimulate you to seek out additional resources on the topic (such as visiting resources like UpToDate or searching the literature)?

a. Never
b. Rarely
c. Some of the time
d. Most of the time
e. Always

Please rate the effectiveness of the following teaching tools in consultation.

**Table:**

	Not effective	Slightly Effective	Moderately Effective	Very Effective	Extremely Effective	N/A -- have not experienced
Telephone interactions						
General text in consult notes						
References in consult notes						
In-person casual interactions						
Being given articles/handouts						
Organized talks (ex. chalk talks, short formal discussions)						

Below is a list of potential barriers to teaching in the consultation process. Please indicate your agreement/disagreement that these factors influence teaching of residents by fellows.

**Table:**

	Strongly Disagree	Disagree	Neither Agree nor Disagree	Agree	Strongly Agree
Lack of time to teach					
Lack of training as teachers					
Lack of interest in teaching					
Lack of knowledge on the subject matter					
Perception of residents as too busy for learning from fellows					
Perception of residents as not interested in learning from fellows					
Amount of teaching fellows must provide to residents rotating on their own service					
Difficulty in locating residents for in-person interactions					
Duty-hour restrictions for residents					
The availability of technological resources (such as UptoDate) for education on the wards					

Do you have any thoughts (good or bad) you would like us to know about the education process within inpatient consultation? If so, please feel free to write them here.

What year of residency are you in?

a. PGY-1 (Intern)
b. PGY-2 (Junior Resident)
c. PGY-3/4 (Senior Resident/Chief Resident)

What is your gender identity?

a. Male
b. Female
c. Other

What is your intended career plan?

a. Primary care (without fellowship)
b. Fellowship -- please indicate ____________________
c. Hospitalist
d. Mix Primary Care/Outpatient Medicine and Hospitalist
e. Unknown
f. Other ____________________
