# Supplementary material for: Education in consultation: A comparative survey-based assessment of perceived educational effectiveness within a university and community-based internal medicine residency
Source: MedEdPublish (2016). 2017 Sep 4;6:153. [Version 1] doi: 10.15694/mep.2017.000153 (PMC10885288; doi:10.15694/mep.2017.000153)
Supplement: Peer review history

# Reviewer report: Reviewer response for version 1

**DOI:** 10.21956/mep.19338.r28245

**Published:** 2017-09-10

**Authors:** Trevor Gibbs

**Affiliations:** 1 AMEE

This review has been migrated. The reviewer awarded 4 stars out of 5 There are some things that we take for granted in medical education; one of them being the expectation that learning takes place when students observe / participate in a real live consultation. I suppose we also take for granted that consultations in different settings provide and support different learning opportunities. This paper touches on both instances and as such is an interesting paper to read, although I think that it forms the basis of a deeper piece of research.We often hear that faculty want to teach / want students to learn within the consultation and then argue that there is never enough time because of the conflict between teaching and service provision, but are we just making an excuse for not factoring in that time and raising the importance of education - if something needs to be done we find ways of doing it!I look forward to the authors taking this piece of the educational jigsaw forward but in the meantime I think it is an interesting paper if only for its pointer towards a need for change

Reviewer Expertise:

NA

No decision status is available

# Reviewer report: Reviewer response for version 1

**DOI:** 10.21956/mep.19338.r28246

**Published:** 2017-09-05

**Authors:** Tan Nguyen

**Affiliations:** 1 Deakin University

This review has been migrated. The reviewer awarded 3 stars out of 5 This paper provides insight into educational practice at a local context. I am curious to know what hypothesis was made when comparing the effectiveness of educational practice at the two teaching environments. It would be beneficial to include the perspective of the clinical educators and/or clients/patients as well on teaching effectiveness. I think greater specificity is required to define what is teaching effectiveness since this definition could vary significantly between participants of the study, by considering discrete educational outcomes or competencies. For example, professionalism, empathy, etc. Reporting on how the interactions were also assessed would provide value such as whether it was formal/informal, descriptive comments, numerical scores, competency based assessment, etc.

Reviewer Expertise:

NA

No decision status is available
